# Generation of silent synapses in dentate gyrus correlates with development of alcohol addiction

**DOI:** 10.1038/s41386-018-0119-4

**Published:** 2018-06-15

**Authors:** Anna Beroun, Maria Nalberczak-Skóra, Zofia Harda, Małgorzata Piechota, Magdalena Ziółkowska, Anna Cały, Roberto Pagano, Kasia Radwanska

**Affiliations:** 1 Department of Molecular and Cellular Neuroscience, the Nencki Institute of Experimental Biology of Polish Academy of Sciences, ul. L. Pasteura 3, Warsaw, 02-093 Poland; 2 Present Address: Department of Molecular Neuropharmacology, Institute of Pharmacology, Polish Academy of Sciences, ul. Smętna 12, Krakow, 31-343 Poland

## Abstract

The brain circuits and synaptic processes that underlie alcohol addiction are currently the subject of intensive research. Here we focus on hippocampal circuitry and show that chemogenetic inhibition of dentate gyrus (DG) during presentation of alcohol-associated cues has long-lasting effects on mice behavior. DG inhibition enhances alcohol seeking and drinking, suggesting that DG regulates addiction-related behaviors. To test this hypothesis, we perform whole-cell patch-clamp recordings from the granule cells of DG and look for electrophysiological correlates of alcohol addiction. We observe that presentation of alcohol-associated cue light that induces relapse to alcohol-seeking results in generation of silent synapses, that lack functional AMPA receptors. Furthermore, using human criteria of addiction, we differentiate mice controlling their alcohol consumption from those that undergo transition to addiction to discover that the levels of silent synapses induced by alcohol cues are specifically increased in the addicted mice. As the total level of dendritic spines that harbor synapses is constant at this time point, our data indicate that synapses of perforant path to DG are weakened during cue relapse. Finally we demonstrate that, acamprosate, a drug that limits alcohol drinking and seeking in addicts, prevents generation of silent synapses in DG upon presentation of alcohol-associated cues. Altogether, our data suggest that weakening of DG synapses upon cue relapse contributes to persistent alcohol addiction-related behaviors.

## Introduction

Alcohol addiction is a chronic disease characterized by extremely high motivation to drink alcohol, difficulty limiting its use as well as high propensity of relapse [1]. The affected brain circuits and the underlying mechanism of maladaptive brain plasticity for alcohol seeking to become compulsive remains unclear. Increasing number of studies indicates that the hippocampus, in addition to its well-established function in spatial navigation and memory processes [2, 3], is involved in pathophysiology of several psychiatric disorders [4–6], including drug addiction [7]. Exposure to cues and contexts associated with drugs of abuse increases hippocampal activity both in human addicts [8–10] and animals [11, 12]. Inactivation of the hippocampus impairs formation and retrieval of drug-associated memory [13–15], and prevents relapse induced by the drug-associated context [16–18]. Conversely, stimulation of the hippocampus at theta frequency causes relapse to drug seeking [19]. It remains, however unclear, how three sub-regions of the hippocampus (dentate gyrus (DG), CA3, and CA1) contribute to drug-related behaviors.

Here we focus on DG as it is the primary input to the hippocampal circuit [20]. The growing body of evidence implicates DG in coding information related to reward. DG is essential for generating contextual memories of drug-induced reward [21, 22]. The recent studies identified a new type of non-spatial, sparsely active, DG granule cells (DGCs) that fire during reward consumption [23]. Surprisingly, these cells comprise 65% of GC [23], suggesting that participation of DG to reward circuitry has been underappreciated so far. Moreover, dopaminergic stimulation of DG results in a long-term depression of its cortical inputs [24], and DG stimulation results in DA release in the nucleus accumbens [25], indicating complex interactions between DG and ventral tegmental area (VTA). Still the function of DG in the regulation of drug reward-driven behavior and underlying synaptic processes are mostly unknown.

A large number of functional and morphological modifications of the brain circuits induced by addictive drugs have been described [26, 27]. Recently, generation of silent synapses has been discovered as a form of synaptic plasticity linked with addiction [28–30]. Silent synapses are thought to be immature glutamatergic synapses containing stable NMDA receptors, whereas AMPA receptors (AMPARs) are either absent or highly labile [31–33]. De novo generation of silent synapses may create new synaptic contacts when they mature by recruiting new AMPARs. Alternatively generation of silent synapses was linked with weakening and elimination of dendritic spines [34]. Both of these processes remodel neuro-circuits and plausibly shape future, addiction-related behaviors [31, 35, 36]. For example, generation of silent synapses in NAc was implicated in development of drug craving and seeking [30, 37, 38]. Our previous studies showed that silent synapses in the central nucleus of the amygdala are linked with increased motivation for alcohol [39]. It is, however, uncertain whether these alterations occur specifically in individuals that develop addiction or are nonspecific adaptations common to all individuals exposed to drugs. Thus the aims of the current study were to verify the function of DG in the regulation of addiction-related behavior and to test whether synaptic plasticity induced by alcohol differentiates addict or non-addict mice. To achieve these aims we developed a mouse model of alcohol addiction [39–41], based on the human criteria of alcohol dependence defined in Diagnostic and Statistical Manual of Mental Disorders, 4th edition (DSM-IV) [1]. The model allows discerning addict mice showing high voluntary alcohol consumption and high motivation for alcohol, persistent, and compulsive alcohol seeking and taking, as well as high intensity of relapse, from non-addict animals, which control their behavior [41]. Here, used the model to study synaptic plasticity of DG granule cells in mice characterized as addict or non-addict.

## Materials and methods

### Subjects

In all experiments, eight-week-old female C57BL/6J mice were purchased from the Medical University of Bialystok, Poland. We did not use male mice as social hierarchy and aggression between males precludes experiments in the IntelliCages. The mice were housed under a 12 h/12 h light/dark cycle and had access to water and food ad libitum. All experiments were approved by the Animal Protection Act of Poland guidelines and approved by the 1st Local Ethical Committee in Warsaw, Poland (no 438/2013 and 119/2016). All efforts were made to minimize the number of animals used and their suffering.

### Animal model of alcohol addiction

To evaluate mice behavior, according to human criteria of alcohol dependence defined in DSM-IV (2000), we developed a model for the IntelliCages [39–41]. The model included assessment of five addiction-related behaviors: (i) high alcohol consumption during free access periods; (ii) high motivation for alcohol in progressive schedule of reinforcement; (iii) persistence of alcohol seeking even during periods of alcohol non-availability; (iv) alcohol seeking induced by alcohol-associated light cue, as well as (v) excessive alcohol consumption during relapse after alcohol withdrawal. The full description of all phases of the training can be found in the Supplementary Online Materials.

#### Establishment of mouse subpopulations

The addiction index was calculated based on five behaviors: (i) the ratio reached during the motivation test, (ii) persistence in alcohol seeking during the persistence test, (iii) alcohol seeking during the cue-induced relapse, (iv) alcohol consumption during the alcohol relapse, and (v) during all free access periods. An individual was arbitrarily considered positive for an addiction-like criterion when its score in the test was in the uppermost 35% of the population. The scoring allowed us to divide the mice into groups according to the number of fulfilled addiction-like criteria: “Addict” who fulfilled two or more criteria (DSM-IV Development); “Non-addict” who were positive for none of the criteria. Moreover, as the addiction index may neglect mice performance in some tests, we developed addiction score. To calculate addiction score each of the addiction-like behaviors was normalized and summed up to calculate individual addiction score according to formula: AS = Σ(Vi (individual score)−mean(population))/SD(population). This allowed us to distinguish mice, which show consistent behavioral patterns towards alcohol.

### Electrophysiology

Patch-clamp technique was used to analyze silent synapses as previously described [39]. Slices were transferred to the recording chamber, perfused with artificial cerebrospinal fluid (ACSF) solution heated up to 31°C. Stimulating electrode was placed in the perforant path. Granule cells of the upper blade of dorsal DG were identified visually and patched with borosilicate glass capillaries (4–6 MΩ resistance) filled with internal solution (130 mM Cs gluconate, 20 mM HEPES, 3 mM TEA-Cl, 0.4 mM EGTA, 4 mM Na_2_ATP, 0.3 mM NaGTP, and 4 mM QX-314Cl, pH = 7.0–7.1, osmolarity: 290–295 mOsm). Series and input resistances were monitored throughout the experiment. Electrical stimulation was elicited by Transistor-transistor logic pulse every 5 s. Recorded currents were filtered at 2 kHz (npi amplifiers) and digitized at 10 kHz (ITC-18 InstruTECH/HEKA). All recordings were performed in the presence of 50 μM picrotoxin (Abcam) in ACSF, to pharmacologically block inhibitory neurotransmission and focus on excitatory pathway specifically.

Frequency of silent synapses: for assessing the frequency of silent synapses on the neurons in DG, the minimal stimulation protocol was used [28, 42]. The strength of the electric pulse was decreased and adjusted to obtain both responses and failures of AMPAR- and NMDAR-mediated EPSCs, recorded at − 60 and + 45 mV, respectively. The full description of procedure can be found in the Supplementary Online Materials

### DiI staining

The hemisphere was postfixed in 1.5% paraformoldehyde (PFA) for 2 h at room temperature (RT) and transferred to ice-cold phospate-buffered saline (PBS) for another 10–20 min. The brains were cut into 130-μm-thick slices using a vibratome (Leica) and kept in RT PBS for 1 h. Six slices containing dorsal hippocampus per animal were labeled by tungsten particles (Bio-Rad, 156-2268) coated with the lipophilic dye (1,1-dioctadecyl-3,3,3,3-tetramethylindocarbocyanine perchlorate, DiI; Life Technologies, D-282). The particles were delivered by gene gun (Bio-Rad) through nylon filter (Merc Millipore, 10 μm, NY1004700). DiI bullets were prepared as described previously (19). Next, the slices were incubated in RT PBS overnight to allow for dye diffusion throughout the cell membrane. After that, slices were transferred to 4% PFA for 2 h, washed in PBS, mounted on slides and covered with DAPI Fluoromount-G (Southernbiotech, 00-4959-52). Z-stacks of confocal images of the 5–7 dendrites per animal from the middle molecular layer of the upper blade of dorsal DG were acquired using Zeiss Spinning Disc confocal microscope (lens: 63 × oil immersion, pixel size: 132 × 132 × 260 nm). Neurons of immature morphology (fewer dendrites, very sparse dendritic spines) were excluded from the analysis. Images were deconvoluted in Autoquantx2 and maximal projections were obtained in ImageJ. Spine clustering into thin, mushroom, and stubby was automatically performed using NeuronStudio software.

### DREADD experiment

Mice were bilaterally injected into dorsal DG (stereotactic coordinates from bregma: ML, ± 1.0 mm; AP, −2.0 mm; DV, −2.0 mm) adeno-associated viral vectors (AAV, serotype 1 and 2) coding inhibitory and activatory designer receptors exclusively activated by designer drugs (DREADD) (9) (pAAV-hSyn-HA-hM4D(Gi)-mCherry, Addgene Plasmid #50475; pAAV-hSyn-HA-hM3D(Gq)-mCherry, Addgene Plasmid #50474) (0.5 μl/ site, viral titer 1.30-1.9 × 10^9^/μl) or control mCherry (pAAV-CaMKIIa-mCherry (0.5 μl/ site, viral titer 1.35 × 10^9^/μl).

After surgery, animals were trained in the IntelliCages as described above and in Supplementary Online Materials.

### Acamprosate experiment

Ten days after the end of the tests for addiction-related behaviors, mice were trained to drink daily 500 licks of acamprosate calcium (Campral) (0.04% solution in tap water, in one bottle in the water corner) from the water corner before getting access to any other liquid (all doors except for the acamprosate were inactive). That resulted in consumption of ~ 250 mg of acamprosate/kg/day. This dose was previously shown to reduce alcohol consumption in rats after extended access to alcohol (Spanagel et al. 1996). In the control groups, mice were forced to drink 500 licks of water from one corner. After 15 days of acamprosate treatment (days 111–115) mice had 7-day withdrawal (days 126–132), followed by 90-minut cue relapse.

## Results

### The effect of chemogenetic manipulation of DG on alcohol seeking and drinking

**Fig. 1:**
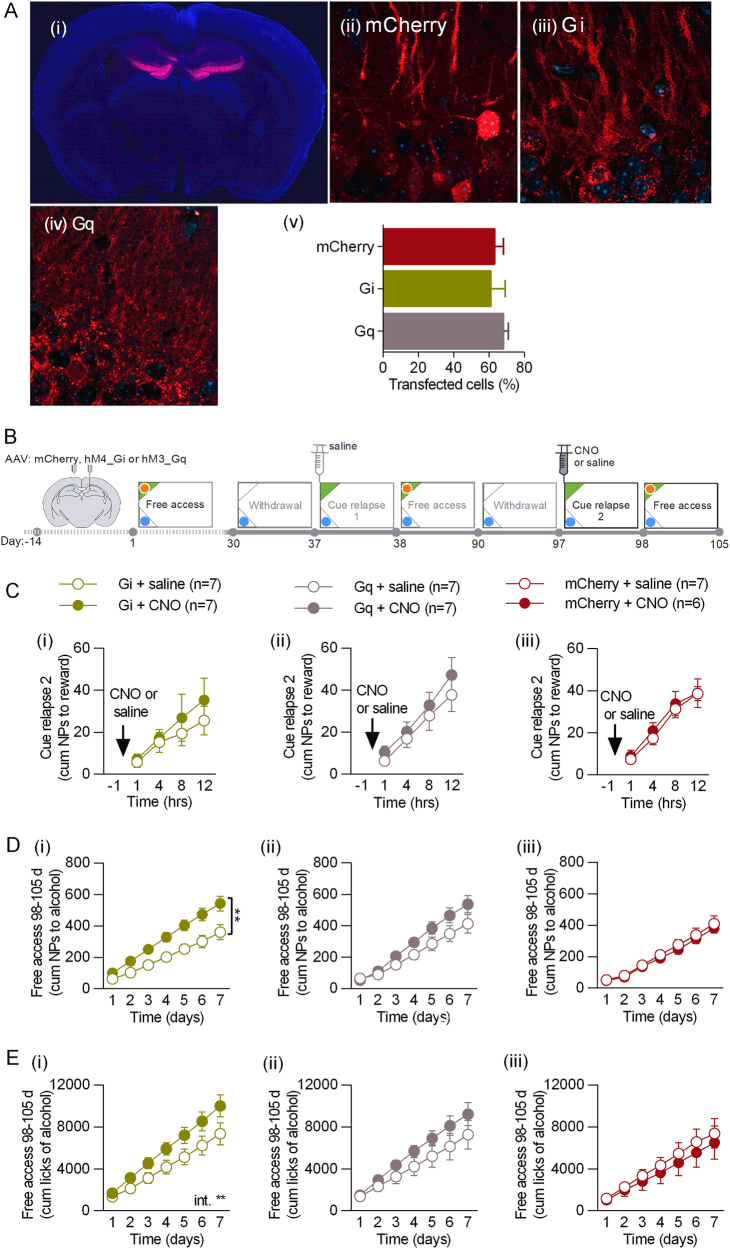
Chemogenetic inhibition of dorsal DG during cue relapse enhances alcohol seeking and drinking. **a** Expression of AAV1/2 encoding DREADDs. (i) Transfection area in dorsal DG. Expression of the control (AAV-CaMKII-mCherry) (ii), inhibitory (AAV-hSyn-hM4D(Gi)-mCherry) (iii), and activatory DREADDs (AAV-hSyn-hM3D(Gq)-mCherry) (iv) in cell bodies and dendrites of the granule cells (upper blade). (v) Quantification of transfected cells in dorsal DG. **b** Experimental timeline. **c** CNO had no effect on alcohol seeking during 2nd cue relapse in any of the experimental groups. **d** CNO enhanced alcohol seeking during period of free access to alcohol that followed cue relapse in mice expressing Gi (i), but not Gq (ii), or mCherry (iii). **e** CNO increased alcohol drinking during period of free access to alcohol in mice expressing Gi (i), but not Gq (ii), or mCherry (iii)

To verify the function of DG in regulation of alcohol addiction-related behaviors, we chemogenetically manipulated the activity of DG of C57BL/6J female mice by expression of adeno-associated viral vectors, serotype 1 and 2 (AAV1/2), encoding inhibitory designer receptors exclusively activated by designer drugs (DREADD) (AAV-hSyn-hM4D(Gi)-mCherry), or activatory DREADDs (AAV-hSyn-hM3D(Gq)-mCherry) [43], or the control virus (AAV-CaMKII-mCherry) (Fig. 1a). Post-training analysis of the hippocampal tissue revealed that DREADDs were expressed in ~ 60% of the granule cells of DG (both in cell bodies and dendrites) (Fig. 1a).

Two weeks after the surgery and viral infection, mice underwent alcohol self-administration training in the IntelliCages [39, 40]. Groups of 14 mice per cage were trained to drink 12% ethyl alcohol (vol/vol). Each cage had two active operant chambers (corners) (Fig. 1b), each visit to a reward corner resulted in presentation of green light (cue) and each nose-poke allowed mice to drink alcohol for 5 s. In a water corner, no specific cue was presented and all mice had unlimited access to water. Free access to alcohol was intermitted by two 7-day withdrawal periods (the reward corner was inactivated) followed by a 24-hour cue relapses induced by the presentation of alcohol-associated cue light in the reward corner without any alcohol available (Fig. 1b). No difference in alcohol seeking between the experimental groups was observed during the first cue relapse and the following period of free access to alcohol (Fig. S1). Twenty minutes before the second cue relapse, mice received i.p. injection of clozapine N-oxide (CNO, 0.5 mg/kg) to activate DREADDs, or saline (Fig. 1b, day 97). We manipulated DG activity at this time point of the training as our data showed that specifically presentation of alcohol-associated cues affects plasticity of DG (Fig. S2). Surprisingly, no effect of CNO injection on alcohol seeking was observed during the cue relapse in any of the experimental groups (repeated measures analysis of variance (RM ANOVA) for Gi: F(1, 12) = 0.3927, *p* = 0.562; Gq: F(1, 11) = 0.5983, *p* = 0.455; mCherry: F(1, 11) = 0.162, *p* = 0.694) (Fig. 1c). We found, however, that inhibition of DG granule cells by CNO in mice expressing inhibitory DREADD receptor hM4D(Gi) had long-lasting effects on alcohol seeking and consumption during the following period of free access to alcohol. The mice performed more nosepokes to the cued corner (RM ANOVA for Gi: F(1, 11) = 10.60, *p* = 0.007) (Fig. 1d.i) and drank more alcohol (F(1, 11) = 3.402, *p* = 0.092, CNO **×** time: F(6, 66) = 3.159, *p* = 0.008) (Fig. 1e.i), than mice injected with saline. This effect of CNO was not observed, neither in the control mice expressing activatory DREADD hM3D(Gq) (NPs: F(1, 12) = 2.660, *p* = 0.129; consumption: F(1, 12) = 1.488, *p* = 0.246) (Fig. 1d–e.ii) nor in the mice expressing mCherry (NPs: F(1, 11) = 0.256, *p* = 0.622; consumption: F(1, 11) = 0.283, *p* = 0.615) (Fig. 1d–e.iii).

### Electrophysiological characteristics of addicted and non-addicted mice

In the following step we determined whether the function of DG is affected when mice are trained to drink and look for alcohol in the IntelliCages. To this end we applied recently developed model of alcohol addiction, which allows to discern mice controlling their alcohol consumption from those that undergo transition to addiction [39–41]. As chemogenetic inhibition of DG enhanced alcohol seeking and consumption, we hypothesized that increased alcohol seeking and drinking in addict mice is linked with weakened DG synapses.

**Fig. 2:**
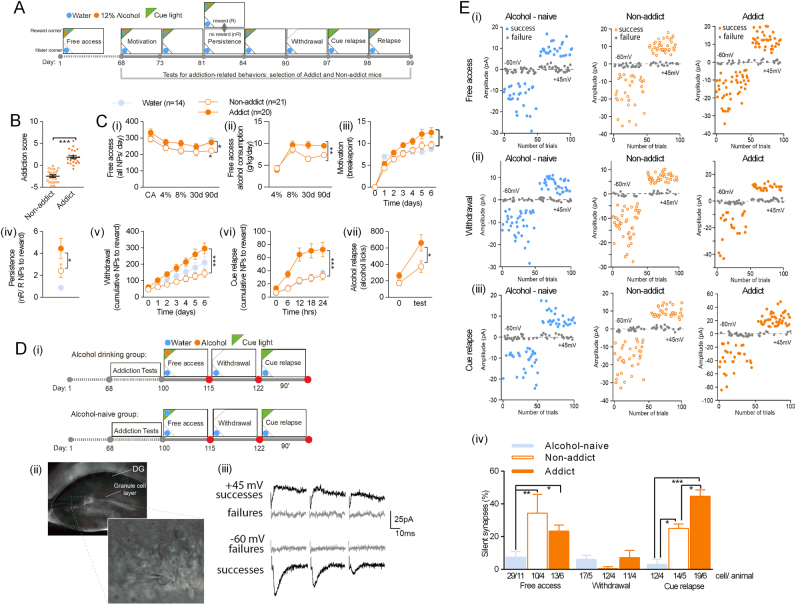
Addicted mice have more silent synapses in DG during cue relapse than non-addict mice. **a** Experimental timeline and IntelliCage setups for alcohol drinking and alcohol-naive mice. Mice went through tests measuring addiction-related behaviors: motivation, persistence, withdrawal, cue relapse, and alcohol relapse spaced by periods of free access to alcohol, to identify addict and non-addict animals. **b** Addicts had significantly higher addiction score than non-addicts (t(27) = 6.844, *p* < 0.0001). **c** Mice performance during the training. (i) Addict and non-addict mice did not differ in nose-poke activity during the cage adaptation (CA) and initial 30 days of free access to alcohol (30d). (ii) They also did not differ in alcohol consumption during initiation of alcohol consumption (4 and 8%). Later (90d) addicts, as compared with non-addict mice, (i) performed more nosepokes to cage corners, and (ii) consumed more alcohol. They also (iii) reached higher breakpoint during Motivation tests, (iv) showed higher increase of reward nosepokes during non-rewarded (nR) phases as compared to rewarded (R) phases during persistence tests; (v) performed more nosepokes to the reward corner during withdrawal; (vi) performed more nosepokes to the reward corner during presentation of alcohol predicting cue; (vii) drank more alcohol during 12 h of relapse (test) as compared with non-addicts, and to the last 12 h of the last day of free alcohol access period (“0”). **d** Electrophysiological analysis of the granule cells in dorsal DG. (i) Experimental timelines and cage setups for alcohol drinking and alcohol-naive mice. Mice were killed during period of free access to alcohol (day 115), withdrawal (day 122) or cue relapse (+90’). (ii) Recording electrode in dorsal DG. Stimulating electrode was in perforant path. (iii) Example EPSCs (successes and failures) elicited by minimal stimulations at +45 mV (top) and −60 mV (bottom). Frequency of successes and failures was used to calculated % of silent synapses (see Materials and Methods for details). **e** Electrophysiological analysis. Trial plots of EPSCs elicited by minimal stimulations at +45 and −60 mV from alcohol-naive, non-addict and addict mice killed (i) during free alcohol drinking, (ii) withdrawal, and (iii) cue relapse, and mice drinking water. (iv) Frequency of silent synapses was increased in alcohol-drinking mice (both addict and non-addict) as compared to alcohol-naive animals during free access to alcohol, and addict mice as compared to non-addict and alcohol-naive mice during cue relapse *p < 0.05, **p < 0.01 by Tukey’s multiple comparisons test

After 68 days of unlimited alcohol self-administration, behaviors that resemble the hallmarks of addiction according to DSM-IV [1] were tested (Fig. 2a): (i) The subject has an extremely high motivation to take the drug, with activities focused on its procurement and consumption. We assessed high motivation for alcohol in progressive schedule of reinforcement in which the number of responses (ratio) to obtain access to alcohol progressively increases within the test. We measured the breakpoint, the last ratio completed, which is considered a reliable index of the motivation for the drug [44] over 7 days of the test. (ii) The subject has difficulty stopping drug use and/or limiting drug intake. These traits were operationalized as persistence in alcohol seeking in persistence test and during alcohol withdrawal as they were previously used to measure addiction-like behaviors in rats and mice [44]. We measured the number of nosepokes performed to the reward corner during periods of alcohol non-availability, as compared with periods when the reward corner was active, in persistence test, and nosepokes performed to the reward corner during 7-day withdrawal. (iii) High propensity to relapse. Again, we measured alcohol seeking during relapse induced by alcohol-associated cue light and excessive alcohol consumption during alcohol relapse after withdrawal. The control, alcohol-naive mice had been tested in the same way as alcohol-exposed mice, however, they had only water in both cage corners during the whole training. Mice performance during all tests was used to indicate addicted and non-addicted individuals. Addicted alcohol drinkers were identified as those, which were positive for at least two addiction-related criteria (top 35% of the population in at least two tests), and non-addict drinkers, which were positive for none of the criteria (for details on how addict and not-addict mice were identified, see Materials and Methods). We also summed up the normalized scores of all tests to obtain addiction score for each animal. Addicted mice had higher total addiction score (t(27) = 6.844, *p* < 0.0001) (Fig. 2b) and higher performance in all tests as compared with non-addicted mice and alcohol-naive mice (Fig. 2c) (motivation tests: F(2, 45) = 3.235, *p* = 0.048, persistence: *U* = 86, *p* < 0.016; withdrawal: F(1, 43) = 12.59, *p* = 0.001, cue relapse F(1, 48) = 13.32, *p* < 0.001; alcohol relapse: F(1, 38) = 5,472, *p* = 0.024), indicating that the measured behavioral features describe consistent phenotype resembling human disorder. Interestingly, although addict and non-addict mice also differed in general activity and alcohol consumption, the difference developed over time and was not observed during 5-day adaptation to the cage (CA), and initiation of alcohol drinking (4 and 8%) (Fig. 2c.i&ii) (all NPs: t(39) = 3.4303, *p* = 0.001; alcohol consumption: F(1, 39) = 6.296, *p* = 0.016). Addicts and non-addicts also did not differ in alcohol preference (Fig. S3).

Next, the mice continued the training (Fig. 2d) and we looked at the electrophysiological differences between alcohol-naive, addicted, and non-addicted mice. We investigated how the extended alcohol drinking affects synaptic plasticity in DG by performing whole-cell voltage clamp recordings from the granule cells of the upper blade of DG, whereas stimulating the perforant path (Fig. 2d, c.ii). Following the observation that chemogenetic inhibition of DG activity enhanced subsequent alcohol consumption and seeking, we measured the content of AMPAR-silent synapses [45, 46], as their appearance is a potential mechanism to weaken basal synaptic transmission. Silent synapses were previously linked with drug craving and addiction in multiple experimental paradigms [28–30, 34]. Moreover, our previous findings show a robust increase of silent synapse number after alcohol consumption in the IntelliCages [39]. Using the minimal stimulation protocol [42] we recorded the frequency of instances when minimal stimulation failed to elicit changes in EPSCs higher than five pA-baseline noise (failures) (Fig. 2d.iii). This was used to calculate % of silent synapses (see Supplementary Materials and Methods for details of the protocol). When alcohol-naive, non-addict, and addict mice were killed during period of free access to alcohol (Fig. 2d.i, day 115) we observed increased frequency of silent synapses both in non-addict and addict drinkers, as compared with the alcohol-naive mice (Fig. 2e) (addiction score: F(2, 37) = 12.92, *p* < 0.001; time: F(2, 37) = 12.47, p < 0.001), suggesting that generation of silent synapses during alcohol consumption was not specific to the severity of alcohol addiction. After 7-day withdrawal (Fig. 2d.i, day 122), the levels of silent synapses were very low (<5%) for all experimental groups and again no significant differences between the experimental groups were found (Fig. 2e). Finally, after 90-min presentation of alcohol-associated cue light (Fig. 2d, day 122 + 90’), we observed a significant increase in the frequency of silent synapses in addict drinkers, as compared with non-addict mice and alcohol-naive controls (Fig. 2e).

**Fig. 3:**
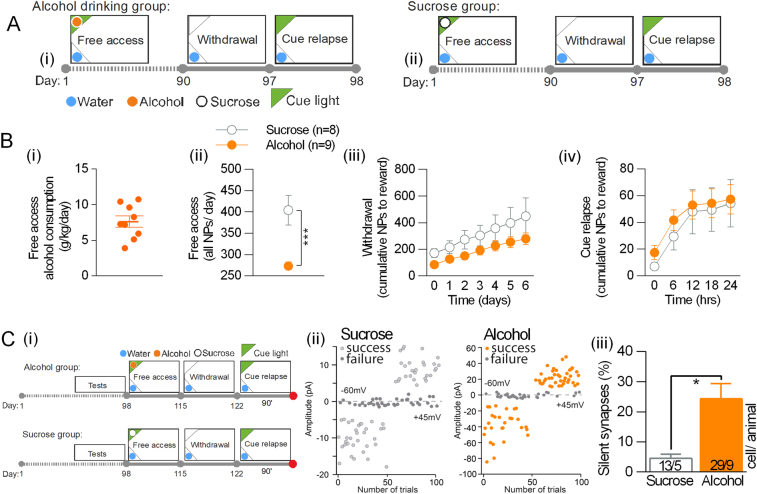
Sucrose-associated cues do not generate AMPAR-silent synapses in DG. **a** Experimental timeline and IntelliCage setups for alcohol drinking (i) and sucrose drinking mice (ii). **b** Mice performance during the training. (i) Alcohol consumption in the group of alcohol drinking mice. (ii) Sucrose mice performed more nosepokes to cage corners during the free access period, as compared with alcohol drinking mice. They were, however, as active as alcohol drinking mice during withdrawal (iii), and cue relapse (iv). **c** Electrophysiological analysis of the granule cells in dorsal DG. (i) Experimental timeline. Mice were killed after cue relapse (+90’). (ii) Trial plots of EPSCs elicited by minimal stimulations at +45 and −60 mV from alcohol and sucrose drinking mice killed during cue relapse. (iii) Frequency of silent synapses was increased in alcohol drinking mice as compared to sucrose drinking animals

In the following experiment we tested whether silent synapses in DG are specific for alcohol-associated cues or are generated during presentation of other salient cues as well. To this end, mice were trained to drink 5% sucrose or 12% alcohol for 90 days (Fig. 3a). During that time, mice drinking sucrose were more active than mice drinking alcohol, measured as the number of nosepokes performed to the active corners (Fig. 3b.ii) (t(26) = 5.09, *p* < 0.0001), as they drank much more liquids. They performed, however, the same number of nosepokes to the cued corner during withdrawal (Fig. 3b.iii) and cue relapse (Fig. 3b.iv) (RM ANOVA withdrawal: F(1, 9) = 2.805, *p* > 0.05; cue relapse: F(1, 9) = 0.237, *p* > 0.01), indicating that sucrose-, as much as alcohol-associated corner and cue light, gained rewarding properties. To measure the levels of silent synapses, alcohol and sucrose drinking mice were killed after 90 min of the cue relapse (Fig. 3c.i, day 122 + 90’). The level of silent synapses in the sucrose mice was significantly lower than in the alcohol drinking mice (~ 4% vs 24%) (Fig. 3c.ii) (t(13) = 2.68; *p* < 0.05). This finding indicates that generation of silent synapses in DG during cue relapse is specific for alcohol-associated cues and does not result only from intensive reward seeking.

### Addiction score affects density of dendritic spines in DG

**Fig. 4:**
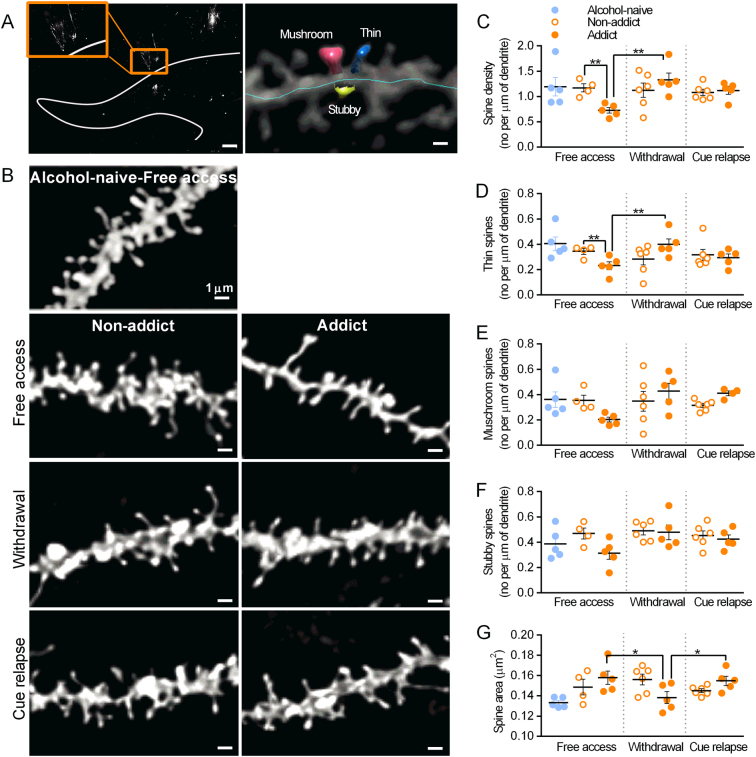
Density of dendritic spines in DG in addict mice is regulated during free access to alcohol and withdrawal, but not cue relapse. **a** DiI staining. (i) The analyzed region of DG. The gray line delineates the outer boundary of the granular layer of DG. Inset: magnification of the stained granule cell. Scale bar, 200 μm. (ii) Exemplary microphotograph of dendritic spines traced with NeuronStudio. Scale bar, 2 μm. **b** Exemplary microphotographs of dendrites from the medial part of the molecular layer of dorsal DG (upper blade) stained with DiI in alcohol-naive, non-addict, and addict animals killed during free access to alcohol, after 7-day withdrawal or 90-min cue relapse. Scale bars, 1 μm. Summary of data showing changes in density of all spines **c**, thin spines **d**, mushroom spines **e**, stubby spines **f**. **g** Summary of data showing changes in size of spines. For all graphs *p < 0.05, **p < 0.01 by Fisher’s test

Formation of silent synapses has been linked with generation of new dendritic spines (after cocaine and nicotine) or rejuvenation of existing synaptic contacts (after morphine) to support addiction-related remodeling of the brain circuits [34, 47]. Alcohol is known to affect morphology and density of dendritic spines [48], it is however unknown how this is linked with electrophysiological properties of the spines and whether addicted individuals differ in this regard from non-addicted ones. We imaged dendritic spines of the granule cells of DG using DiI staining [34], focusing on the dendrites in the medial part of the molecular layer (upper blade) (Fig. 4a, left) that are innervated by the perforant path. Spine density and size, as well as density of spines in three categories (mushroom, stubby, and thin) were automatically assessed using NeuronStudio software (Fig. 4a, right). During the period of free access to alcohol (Fig. 2d.i, day 115) the addict mice had significantly fewer spines than non-addict mice (Fig. 4c) (two-way ANOVA, addiction score: F(1, 25) = 0.620, *p* = 0.438; time: F(2, 25) = 3.529, *p* = 0.044, interaction: F(2, 25) = 5.071, *p* = 0.014). This impairment was reversed during withdrawal (day 122) and no further change in total spine density was detected after 90 min of cue relapse (day 122 + 90’). Detailed analysis of the spines in three shape categories showed that the density of thin spines was decreased during free access to alcohol in addict mice, as compared with non-addicts (Fig. 4d) (addiction score: F(1, 25) = 0.041, *p* = 0.839; time: F(2, 25) = 1.969, *p* = 0.160, interaction: F(2, 25) = 6.115, *p* = 0.007). This was reversed during withdrawal and not affected by cue relapse. No statistically significant change in density of mushroom or stubby spines was observed (Fig. 4e, f) (mushroom, addiction score: F(1, 26) = 0.095, *p* = 0.760; time: F(2, 26) = 1.531, *p* = 0.235, interaction: F(2, 26) = 2.499, *p* = 0.101); Stubby, addiction score: F(1, 26) = 3.938, *p* = 0.057; time: F(2, 26) = 2.443, *p* = 0.106, interaction: F(2, 26) = 1.681, *p* = 0.205). Furthermore, addict mice had bigger spines than the controls during free access to alcohol. Their size decreased during withdrawal and increased during cue relapse (Fig. 4g) (addiction score: F(1, 25) = 0.007, *p* = 0.929; time: F(2, 25) = 0.633, *p* = 0.539, interaction: F(2, 25) = 4.555, *p* = 0.020), suggesting that these changes resulted from disappearance and reappearance of small, thin dendritic spines. Notably, no change in density and size of dendritic spines was observed in non-addict mice during the training. In conclusions, our data show that changes in the density of thin spines, next to the changes of silent synapses, are the hallmark of addict mice.

### The effect of acamprosate on alcohol drinking and seeking, and silent synapses in DG

**Fig. 5:**
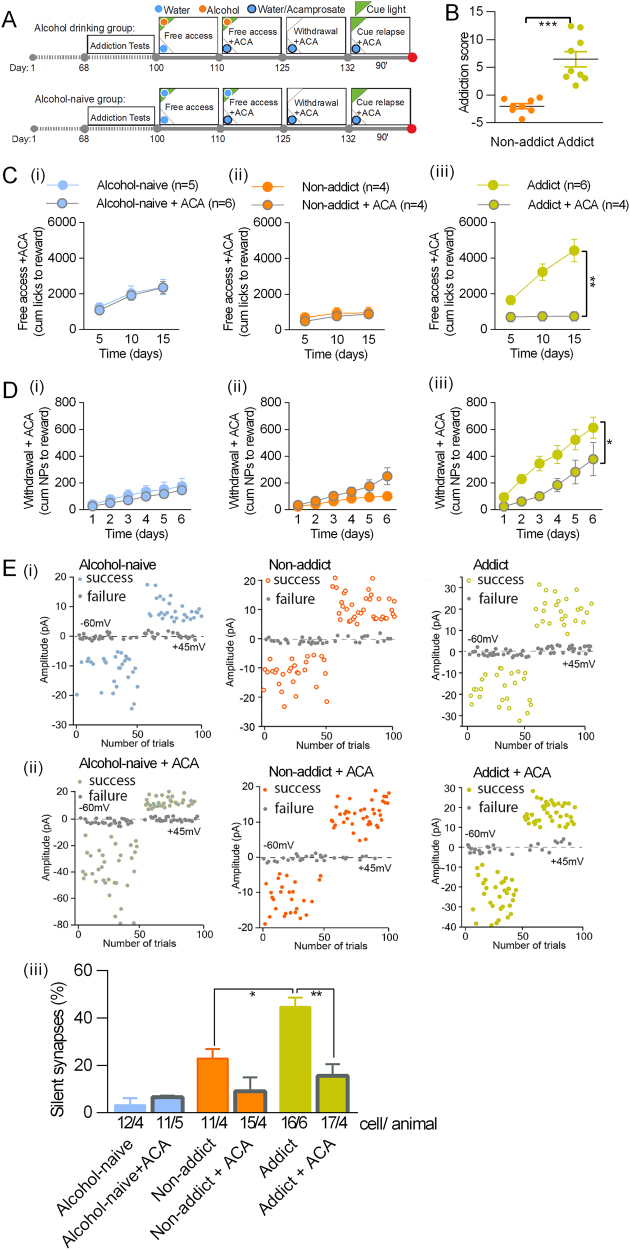
Acamprosate abates generation of silent synapses in dorsal DG during alcohol cue relapse in addict mice. **a** Experimental timeline and IntelliCage setups. Mice went through tests measuring addiction-related behaviors to identify addict and non-addict animals, followed by acamprosate treatment (ACA) from day 110. **b** Addicts, identified during addiction tests, had significantly higher addiction score than non-addicts. **c** Exposure to acamprosate decreased alcohol consumption in addict mice. **d** Exposure to acamprosate decreased alcohol seeking during withdrawal in addict mice. **e** Electrophysiological analysis (day 132 + 90’). Trial plots of EPSCs elicited by minimal stimulations at + 45 and − 60 mV from alcohol-naive (left), non-addict (center), and addict mice (right) without (i) or with acamprosate treatment (ii). (iii) Summary of data showing that acamprosate treatment prevented generation of silent synapses upon cue relapse in addict mice. *p < 0.05, **p < 0.01 by Tukey’s multiple comparisons test

Some of the behavioral manifestations of alcohol addiction, such as alcohol craving and consumption, can be suppressed by acamprosate treatment [49]. Therefore, we determined whether acamprosate can also prevent cue relapse-induced generation of silent synapses in alcohol addict mice. To this end, mice underwent alcohol self-administration training followed by the tests to select addict and non-addict mice (Fig. 5a). After the tests half of the addict and non-addict mice (as well as the alcohol-naive mice) had been given acamprosate in drinking water (250 mg/kg/day, ACA) (Fig. 5a). The rest of alcohol-drinking mice drank only alcohol and water. We observed that acamprosate decreased alcohol consumption during the period of free access to alcohol (Fig. 5c) (alcohol-naive F(1, 9) = 0.077, *p* = 0.786; non-addict: F(1, 6) = 0.227, p = 0.650, addict: F(1, 7) = 18.50, *p* = 0.003) and alcohol seeking during withdrawal of addict mice (Fig. 5d) (alcohol-naive: F(1, 7) = 0.561, p = 0.478; non-addict: F(1, 7) = 3.271, *p* = 0.113, addict: F(1, 6) = 8.686, p = 0.025), but not in non-addict mice. Neither did we observe any effect of acamprosate on the behavior of alcohol-naive mice. Next, the mice were killed after 90 min of the cue presentation to measure the number of silent synapses (Fig. 5a, day 122 + 90’). We did not observe the differences in the behavior of the mice from the experimental groups during this short period (Fig. S4). Consumption of acamprosate prevented, however, formation of silent synapses during presentation of cue light associated with alcohol in addicted mice (Fig. 5e) (addiction: F(2, 19) = 20.65, *p* < 0.0001, acamprosate: F(1, 19) = 15.98, *p* = 0.0008, interaction: F(2, 19) = 8.094, *p* = 0.0029). Acamprosate did not alter silent synapse number neither in alcohol-naive nor non-addict mice.

## Discussion

Our study shows that chemogenetic inhibition of DG during cue relapse has a long-lasting effect on mice behavior enhancing alcohol seeking and consumption. Our finding therefore suggests DG function in regulation of addiction-related behaviors. To test this hypothesis, we employed whole-cell patch-clamp recordings to discover that presentation of alcohol-associated cues that induced relapse to alcohol seeking leads to the generation of AMPAR-silent synapses in the perforant path input to the granule cells of DG. The levels of silent synapses during cue relapse is higher in addicted individuals than non-addict mice. As their generation is not accompanied by generation of new dendritic spines, our data indicate that cue relapse results in weakening of the existing synapses of perforant path to DG, and it is related to the addiction status of an individual. Moreover, acamprosate, a medication that decreases alcohol seeking and consumption, prevents generation of silent synapses in addict mice. Thus, our data reveal the synaptic plasticity of DG induced by alcohol-associated cues, which is specifically enhanced in addict individuals and has a potential for long-lasting control over alcohol seeking and drinking.

### Function of dorsal DG in cue relapse and addiction-related behaviors

The increasing number of studies links hippocampus and its DG with addiction-related behaviors [50]. DG is essential for generation and expression of contextual memories of drug-induced reward [21, 22], and relapse induced by the context associated with the drug [51]. However, recent studies identified a new type of DGCs that fire during reward consumption independently of its spatial location [23], indicating that not only the contextual features of reward are processed in DG. Here, using our previously validated animal model of alcoholism in the IntelliCage [39], we determined the effect of long-term alcohol exposure on synapses in DG. We demonstrated that exposure to alcohol, alcohol withdrawal and alcohol-associated cues (green light) after prolonged withdrawal affect synaptic plasticity of the granule cells of dorsal DG, which is manifested by generation and disappearance of silent synapses [31, 36]. Hence, our data support the notion that dorsal DG is involved in processing information about reward and discrete cues associated with reward [52, 53].

Surprisingly, temporary, chemogenetic inhibition of the granule cells during cue relapse had no effect on the intensity of cue relapse. This observation is in agreement with the earlier observations showing that hippocampus drives relapse induced by drug-associated context rather than discrete cues [54]. To further elucidate the role of DG in alcohol addiction-related behaviors, we took the advantage of the IntelliCage system, which allows for long-term monitoring of mice behavior. We noticed that chemogenetic inhibition of DG during cue relapse had delayed effects on mice behavior and enhanced alcohol seeking and consumption during free access period that followed cue relapse. Our observation agrees with the earlier findings showing that optogenetic manipulation of DG alters animal arousal even after the light delivery is terminated [55]. Thus, our studies suggest that information processed by DG about salient, alcohol-associated cues shapes future behavior triggered by alcohol and alcohol self-administration context. It can also be speculated that chemogenetic inhibition applied during cue-induced relapse induce a long-lasting neural adaptation that alters reward sensitivity. The further studies are required to determine the output and input regions mediating these effects. DG modulates structures involved in exploration, drug seeking, and consumption, such as the lateral septum, nucleus accumbens, and VTA [18, 25], whereas DG activity is modified not only by entorhinal cortex but also dopamine, glutamate, and GABA from VTA [24, 56, 57].

### Remodeling of dendritic spines in DG as a hallmark of addicted individuals

Formation of silent synapses after exposure to drugs of abuse has been linked with development of drug addiction [28–30]. It was, however, never shown whether these modifications occur in all drug-exposed mice or they are specific to those, which undergo transition to addiction. To answer this question, we used previously validated animal model of alcoholism in the IntelliCage [39–41]. Owing to the complexity of addiction process, we tested the mice for several addiction-related behaviors, such as alcohol consumption, motivation to obtain alcohol, persistence in alcohol seeking, and propensity to relapse, to distinguish addicted individuals from those which controlled their behavior. We found that exposure to alcohol increases the frequency of silent synapses in DG, both in addict and non-addict mice. In agreement with previous studies, the silent synapses mature and disappeared from recordings during withdrawal [37], again both in addict and non-addict mice. However, the level of silent synapses generated during cue relapse was significantly higher in the addict mice as compared to the non-addicts. As silent synapses were not observed in mice exposed to water- or sucrose-associated cues, the appearance of silent synapses during cue relapse is not merely triggered by reward seeking or light, but it is specific to alcohol-associated cue light. Overall, our experiments showed that not only exposure to alcohol, but also to alcohol-associated cues results in generation of silent synapses, and this process is a hallmark of addicted individuals. As our data are only correlative, the further research is needed to fully validate the function of silent synapses in DG in regulation of alcohol addiction.

Previous studies found that generation of silent synapses after morphine treatment results from internalization of AMPARs on existing spines followed by spine elimination, whereas after cocaine injections they may appear on new immature spines to remodel brain circuits [34]. Here, we show that in addict mice generation of silent synapses during free access to alcohol is linked with elimination of thin spines, maturation (or elimination) of silent synapses during withdrawal is linked with generation of new spines, whereas formation of silent synapses during cue relapse is accompanied by no noticeable changes in spine density. These data indicate weakening of the perforant path synapses during free access to alcohol and cue relapse, and strengthening of the pathway during withdrawal. At the same time, in non-addict mice the changes of the levels of silent synapses were not accompanied by any noticeable changes in density and size of dendritic spines, indicating that regulation of spine morphology and function may be uncoupled, and depend on the addiction status. The mechanism driving synaptic plasticity of perforant pathway in alcohol drinking mice is unknown, however it can be regulated by dopaminergic transmission from VTA, as previous studies showed that stimulation of dopaminergic VTA-DG projections results in a long-term depression of DG cortical inputs [24].

To further test the function of silent synapses in the development of addiction-related behavior, we used acamprosate, which is the most commonly prescribed drug for the treatment of alcoholism [58]. In animal studies, acamprosate was shown to decrease alcohol consumption in mice and rats, decrease alcohol deprivation effect, and inhibit cue-induced reinstatement of alcohol-seeking behavior in operant conditioning model [59]. Here, we confirm that acamprosate decreases alcohol self-administration and seeking in addict mice. Moreover, we observed that acamprosate precluded generation of silent synapses during cue relapse in addict mice. None of these effects were observed in the non-addict mice or mice trained to drink only water. Accordingly, it is conceivable that acamprosate affects pathophysiological alterations, which drive addiction-related behaviors and provoke generation of silent synapses. Acamprosate produces changes in the brain that mimic the effects of NMDAR antagonism [60]. More recent studies indicate that regulation of alcohol-induced behaviors by acamprosate is, at least partly, mediated by inhibition of mGluR5 signaling [61]. Stimulation of mGluR5 induces LTD [62], as well as AMPARs internalization and silencing [63, 64]. Thus, acamprosate treatment may prevent formation of silent synapses by inhibition of mGluR5-driven internalization of AMPARs. However, to understand in detail the molecular processes regulated by acamprosate, future research is still needed.

In conclusion, our study reveals that inhibition of synaptic transmission in DG granule cells has the potential to drive alcohol seeking and drinking. We further demonstrate that weakening of DG transmission by enhanced generation of silent synapses upon presentation of alcohol-associated cues is specific for the addict mice and can be downregulated by anti-craving medications. Overall, our study suggests the important role of DG in transition to addiction.

## Electronic supplementary material

Supplementary materials
