# Supplementary material for: Generation of silent synapses in dentate gyrus correlates with development of alcohol addiction
Source: Neuropsychopharmacology. 2018 Jun 15;43(10):1989–99. doi: 10.1038/s41386-018-0119-4 (PMC6098144; doi:10.1038/s41386-018-0119-4)
Supplement: Supplementary file 1 — Supplementary materials [file 41386_2018_119_MOESM1_ESM.pdf]

Supplementary materials

Generation of silent synapses in dentate gyrus correlates with development of alcohol addiction

Anna Beroun[1]*, Maria Nalberczak-Skóra[1]*, Zofia Harda[1,2], Małgorzata Piechota[1], Magdalena Ziółkowska[1], Anna Cały[1], Roberto Pagano[1] and Kasia Radwanska[1]

*these authors contributed equally

[1]Department of Molecular and Cellular Neuroscience, the Nencki Institute of Experimental Biology of Polish Academy of Sciences, ul. L. Pasteura 3, Warsaw 02-093, Poland.

[2]currently at Department of Molecular Neuropharmacology, Institute of Pharmacology, Polish Academy of Sciences, ul. Smętna 12, Krakow 31-343, Poland.

Corresponding author: Kasia Radwanska, Ph.D., Laboratory of Molecular Basis of Behavior, the Nencki Institute of Experimental Biology of Polish Academy of Sciences, ul. L. Pasteura 3, Warsaw 02-093, Poland; e-mail: k.radwanska@nencki.gov.pl.

Key words**:** silent synapses, alcohol addiction, dentate gyrus, acamprosate, DREADDs, dendritic spines.

Materials and Methods

Animal model of alcohol addiction in the IntelliCages.

IntelliCages. After 1 week of acclimatization, the mice were injected (s.c.) with unique microtransponders (11.5 mm length, 2.2 mm diameter; Trovan, ID-100) under brief isoflurane anesthesia. The mice were then allowed to recover for 3 days, and the animals with properly located microtransponders were introduced to the IntelliCage system (NewBehavior AG, Zürich, Switzerland) (http://www.newbehavior.com/), 15 animals per system. The IntelliCage consists of a large standard rat cage (20.5 cm high, 40 cm x 58 cm at the top, 55 cm x 37.5 cm at the base). In each corner, a triangular learning chamber is located with two bottles. To drink, only one mouse can go inside a plastic ring (outer ring: 50 mm diameter; inner ring: 30 mm diameter; 20 mm depth into outer ring) that ends with two 13 mm holes (one on the left, one on the right) that provide access to bottle nozzles. Each visit to the corner, nose-poke at the doors governing access to the bottles, and licks were recorded by the system and ascribed to a particular animal.

Mice underwent either short training (to study mice behavior during withdrawal and cue relapse) or full training (to identify addict and non-addict mice in five tests). The *short* training was composed of the following tests: (1) adaptation to a new cage (days 1-4); (2) initiation of alcohol drinking (days 5-17); (3) free alcohol access (days 18-90), (3) withdrawal (days 91-97); (4) cue-induced-relapse (day 98); (5) alcohol relapse (day 99); (6) free alcohol consumption (days 100-115); (7) withdrawal (days 116-122); (8) cue-induced-relapse (day 122+90 min). Each test was programmed and turned on and off automatically, without intrusion of an experimenter, at the beginning of the dark phase.

The *full* training was composed of the following tests: (1) adaptation to a new cage (days 1-4); (2) initiation of alcohol drinking (days 5-17); (3) free alcohol access (days 18-68); (4) motivation to alcohol test (days 69-73); (5) free alcohol consumption (days 74-81); (6) persistence in alcohol

seeking test (days 82-84); (7) free alcohol consumption (days 85-91); (8) withdrawal (days 92-98); (9) cue-induced-relapse (day 99); (10) alcohol relapse (day 100); (11) free alcohol consumption (days 101-115); (12) withdrawal (days 115-122); (13) cue-induced-relapse (day 122).

Adaptation phase. All mice had free access to all bottles with water in both active corners. All doors were open. After 24 hours, when all mice visited and licked from both corners, the doors were closed. Under fixed ratio of reinforcement (FR 1), each nose-poke was rewarded by a 5 second access to the bottles with water.

Initiation of alcohol consumption. During the test, 2 corners were active, each with two bottles available. In one corner, the animals had access to water ("water corner"), and in the other ("reward corner") animals had access to ethanol solution at increasing concentrations (4, 8, and 12% ethanol changed every 3 days, prepared from 96% ethanol and tap water). When alcohol was available, it was signaled by a green light turn on in the "reward corner" each time a mouse entered the corner. All liquids were available under an FR1 schedule. The position of the "reward corner" was altered every 3 days. During *free alcohol access phases* mice had unlimited access to water in one corner and 4, 8 and12% ethanol in the "reward corner" (each concentration tested for 3-5 days). Access to water and alcohol was under FR1. The position of the "reward corner" was altered every 3-5 days to assure that mice followed alcohol in whichever corner it was presented. 12% alcohol was chosen based on maximal alcohol consumption in g/kg/day during initiation of alcohol consumption. Daily alcohol consumption (g/kg/day) was calculated with the following formula: (number of licks of 12% alcohol per day x lick volume x 0.12 x 1 g/ml) / animal weight. To calculate the average lick volume, water consumption (in μl) was measured for 3 consecutive days. The average volume of one lick was measured as total volume consumed /

number of licks. According to these calculations, an average lick volume was established as 1.94 ± 0.2 μl. In the control groups, 5% sucrose (in tap water) or tap water was presented in the reward corner through the whole experiment.

Motivation for alcohol tests. During the test, two corners were active and available to animals. The animals had to perform an increasing number of nosepokes (2, 4, 8, 12, 16, 20, 24, 28, 32, and 36) spaced by less than 1 s during one visit to open the door and be allowed for a 5 s access to the reward bottles. The number of required instrumental responses (nosepokes) increased when an animal performed 10 sets of responses of a given ratio. The tests were terminated when 90% of animals did not change FR level during the last 24 hours. The FR level reached during the test was used as an index of motivation.

Persistence in reward seeking tests. The persistence in reward seeking was measured as a number of reward nosepokes during signaled "no-reward" periods (nR). "Reward" periods (R) were signaled by the green cue light in the reward corner. During the "reward" periods, nosepokes at all doors opened the door for 5 s (FR 1). The "no-reward" periods were signaled by elimination of the green cue light. During the "no-reward" periods, nose-pokes on the reward side were not followed by any scheduled consequences. Each persistence test lasted 3 days, starting at the beginning of the light phase and was composed of six, 6-hour long "reward periods" (R) altered with 6-hour long "no-reward periods" (nR). Number of "reward" nose-pokes performed during the test, as well as ratio of nose-pokes performed during no-reward to reward periods were used as an index of persistence.

Alcohol withdrawal followed by cue and alcohol relapse. The withdrawal periods were signaled as the "no-reward" periods and lasted 7 days. Door to reward was closed and nosepokes to the reward corner were without scheduled consequences. Each withdrawal was followed by a 24-

hour cue-relapse. A green cue light (reward-associated cue) in the reward corner was presented each time a mouse entered the reward corner. However, nose-pokes to the reward doors had no scheduled consequences. Number of nosepokes performed in "reward corner" was used as an index of cue-induced relapse. This test was followed by reward relapse when bottles with reward (alcohol or sucrose) were added into the active "reward corner". During the test each nose-poke into reward door opened the door for 5 s. Amount of reward drank during the first day of relapse (number of licks) was used as an index of relapse.

For the electrophysiological analysis, all mice were trained according to the same protocol till the last free access period. The length of the last alcohol consumption period was adjusted for each mouse (from 15 to 30 days) so only one mouse per day finished the training and was used for electrophysiological recordings. The length of the last free access period was balanced between the experimental groups.

Electrophysiology.

Mice were anesthetized with isoflurane and decapitated. The brains were cut in half. One hemisphere was used for electrophysiological recordings, another for DiI staining. For electrophysiological recordings, acute, 250 μm-thick slices were prepared using Leica VT1000S vibratome in ice-cold NMDG cutting solution (135 mM N-methyl-D-glucamine, 1 mM KCl, 1.2 mM KH_2PO_4, 1.5 mM $MgCl_2$, 0.5 mM $CaCl_2$, 20 mM choline bicarbonate, 10 mM D-glucose (all chemicals purchased from Sigma)). Slices containing hippocampus were collected and transferred to ACSF solution (119 mM NaCl, 2.5 mM KCl, 1 mM NaH_2PO_4, 26 mM $NaHCO_3$, 1.3 mM $MgCl_2$, 2.5 mM $CaCl_2$ and 10 mM D-glucose (all chemicals purchased from Sigma)) at RT and incubated for 1h. All solutions were bobbled with carbogene (95% O2, 5% CO2). Patch-clamp technique was used to analyze silent synapses as previously described (Stefaniuk *et al*, 2017). Slices were transferred to the recording chamber, perfused with ACSF solution heated up

to 31°C. Stimulating electrode (septum theta glass capillary with two chlorinated silver wires) was placed in the perforant path. Granule cells of the upper blade of dorsal DG were identified visually and patched with borosilicate glass capillaries (4-6 MΩ resistance) filled with internal solution (130 mM Cs gluconate, 20 mM HEPES, 3 mM TEA-Cl, 0.4 mM EGTA, 4 mM Na_2ATP, 0.3 mM NaGTP, and 4 mM QX-314Cl, pH = 7.0-7.1, osmolarity: 290-295 mOsm). Series and input resistances were monitored throughout the experiment. Electrical stimulation was elicited by TTL pulse every 5 s. Recorded currents were filtered at 2 kHz (npi amplifiers) and digitized at 10 kHz (ITC-18 InstruTECH/HEKA). All recordings were performed in the presence of 50 μM picrotoxin (Abcam) in ACSF, to pharmacologically block inhibitory neurotransmission and focus on excitatory pathway specifically.

Frequency of silent synapses: for assessing the frequency of silent synapses on the neurons in DG, the minimal stimulation protocol was used (Huang *et al*, 2009; Liao *et al*, 1995). The strength of the electric pulse was decreased and adjusted to obtain both responses and failures of AMPAR- and NMDAR-mediated EPSCs, recorded at -60 and +45 mV respectively. The noise level of our recording setup allows to clearly distinguish responses as small as 5 pA above the baseline. This is a crucial matter, as successes and failures are distinguished visually. During data analysis, the experimenter was blinded to the experimental groups. The percentage of silent synapses was calculated from the following equation:

% silent synapses $=1-\ln F_{-60mV}/\ln F_{+45mV}$,

where F_{-60mV} and F_{+45mV} are failure rates at -60 mV and +45 mV respectively.

DREADD experiment.

Mice were anaesthetized with isoflurane (5% for induction, 1.5-2.0% after) in the stereotaxic frame for the entire surgery and their body temperatures were maintained using a heating pad. We bilaterally injected into dorsal DG (stereotactic coordinates from bregma: ML, ±1.0 mm; AP, -2.0mm; DV, -2.0mm) adeno-associated viral vectors (AAV, serotype 1 and 2) coding inhibitory and activatory designer receptors exclusively activated by designer drugs (DREADD) (9) (pAAV-hSyn-HA-hM4D(Gi)-mCherry, Addgene Plasmid #50475; pAAV-hSyn-HA-hM3D(Gq)-mCherry, Addgene Plasmid #50474) (0.5 μl/ site, viral titer 1.30-1.9 $x10^9$/μl) or control mCherry (pAAV-CaMKIIa-mCherry (0.5 μl/ site, viral titer 1.35 $x10^9$/μl). The viruses were prepared by Animal Model Core Facility at Nencki Institute. The viruses were infused by a glass capillary (disposable BLAUBRAND micropipettes, intraMark) mounted to the custom-made pump attached to the Digital New Standard Stereotaxic system from Stoelting (51500D).

After surgery, animals were allowed to recover for 14 days before the training in the IntelliCages. After the initial period of free access to alcohol (day 1-30), the training was composed of the following phases: withdrawal 1 (day 31-37); cue relapse 1 (day 38); free access to alcohol (day 39-90); withdrawal 2 (day 91-97) and cue relapse 2 (day 98); free access to alcohol (day 99-105). Animals were injected with saline 20 minutes before the first cue relapse. Next, mice were injected with CNO (0.5 mg/kg in saline) or saline 20 minutes before second cue relapse. Mice were split into CNO and saline groups, based on their performance during the cue relapse 1 (alcohol seeking during the first cue relapse did not differ between the groups).

At the end of the behavioral training, mice were anesthetized and perfused (PBS/4%PFA). Next the brains were immersed in antifreeze solution (15% sucrose, 30% ethylene glycol in PBS with NaNO3) and cut on a cryostat (Leica CM1850) in 40-μm thick sections. Sections from dorsal hippocampus were mounted on slides and covered with DAPI Fluoromount-G

(Southernbiotech, 00-4959-52). Zeiss Spinning Disc confocal microscope (lens: 63x oil immersion, pixel size: 132x132x260 nm) was used to photograph the dorsal hippocampus and assess the extent of the expression of the viruses. Due to the lack of visible viral expression, one animal was excluded from the behavioral analysis.

Immunostaining

Mice were anesthetized (i.p. sodium pentobarbital; 50 mg/kg) and perfused transcardially with phosphate-buffered saline (PBS (Medicago), 3 min at RT), followed by 4 % paraformaldehyde (PFA, Sigma-Aldrich), 6 min at RT. Brains were removed and placed in 4% PFA at 4° C for 24 hours and next in 30% sucrose at 4° C until sunk. Coronal sections (40 µm) were cut on cryostat (Cryostats Leica CM1950) and stored at -20° C in PBSAF [PBS, 20% sucrose (Sigma–Aldrich), 15% ethylene glycol (Sigma–Aldrich), 0.05% NaN_3 (Sigma–Aldrich)]. The slices were incubated overnight at 4°C with primary antibody directed against P-T286-CaMKII (R&D Systems, PPS002, 1:500) and PSD95 (Millipore, MAB1598, 1:500) in 5% normal donkey serum in 0.3% Triton in PBS. Next day slices were washed in 0.3% Triton X-100 in PBS and incubated with secondary antibody bound with Alexa Fluor 555 (Invitrogen, A21447, 1:500), or Alexa Fluor 647 (Invitrogen, A31570. 1:500). Finally, the sections were washed in PBS and mounted on slides.

Microscopy and image analysis

The immunostaining was photographed with Leica SP5 microscope (63x oil immersion objective and 1.66x digital zoom), equipped with digital camera. Every sixth section through DG (approx.. 6 sections per animal) were photographed bilaterally with the same microscope settings. Images were analyzed using Fiji software. Intensity of the staining was measured using mean grey value

parameter (MGV) for 8-bit images ranging from 0 to 255. All microphotographs were analyzed by a person blind to the experiment groups.

Data analysis.

The results are expressed as mean ± SEM. Differences between groups were considered significant at $p < 0.05$. The statistical analysis of data was performed in GraphPad Prism. The information on statistical tests is in figures legends.

Results

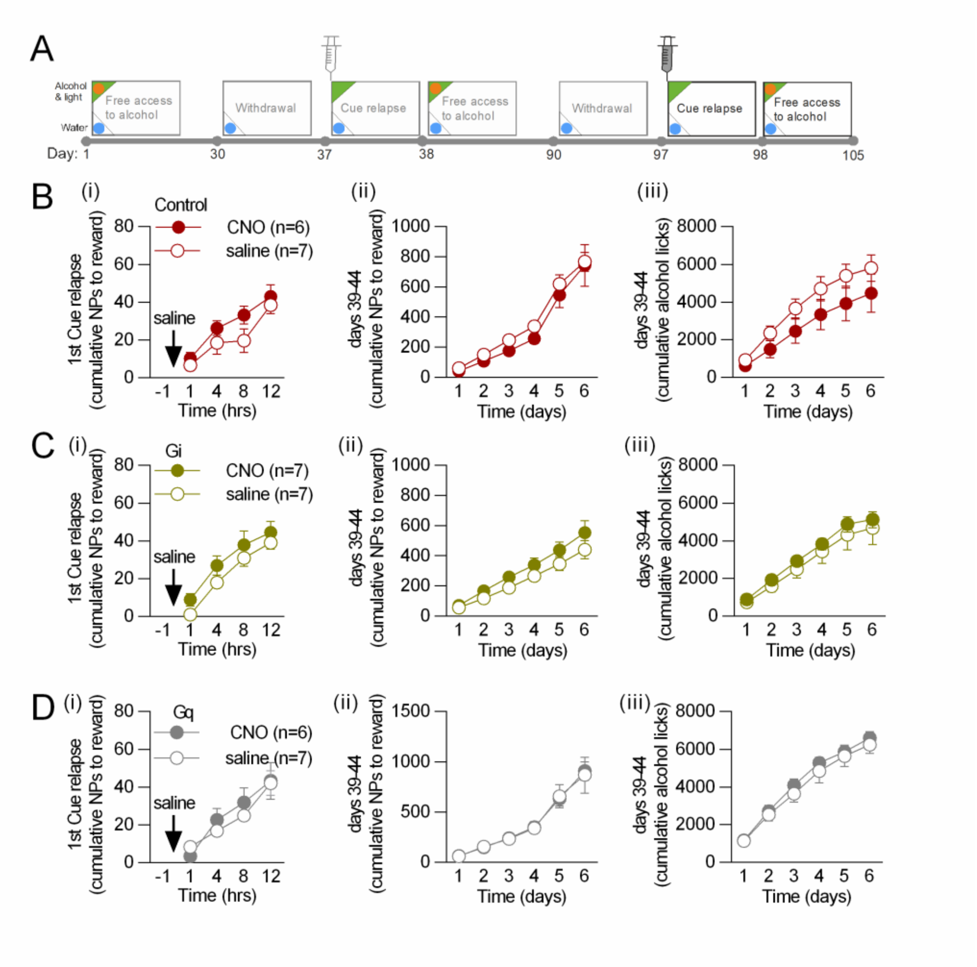

Supplementary Figure 1. DREADD expression had no effect on mice behaviour during the first cue relapse and the following period of free access to alcohol.

(A) Experimental timeline. Before the first cue relapse all mice were injected with saline. “CNO” groups were injected with CNO only before 2nd cue relapse (see Fig. 3).

(B) There was no difference between “CNO” and “saline” groups of mCherry-expressing mice in alcohol seeking during the first cue relapse (i); and the following period of free access to alcohol (ii-iii).

(C) There was no difference between “CNO” and “saline” groups of Gi-expressing mice in alcohol seeking during the first cue relapse (i); and the following period of free access to alcohol (ii-iii).

(D) There was no difference between “CNO” and “saline” groups of Gq-expressing mice in alcohol seeking during the first cue relapse (i); and the following period of free access to alcohol (ii-iii).

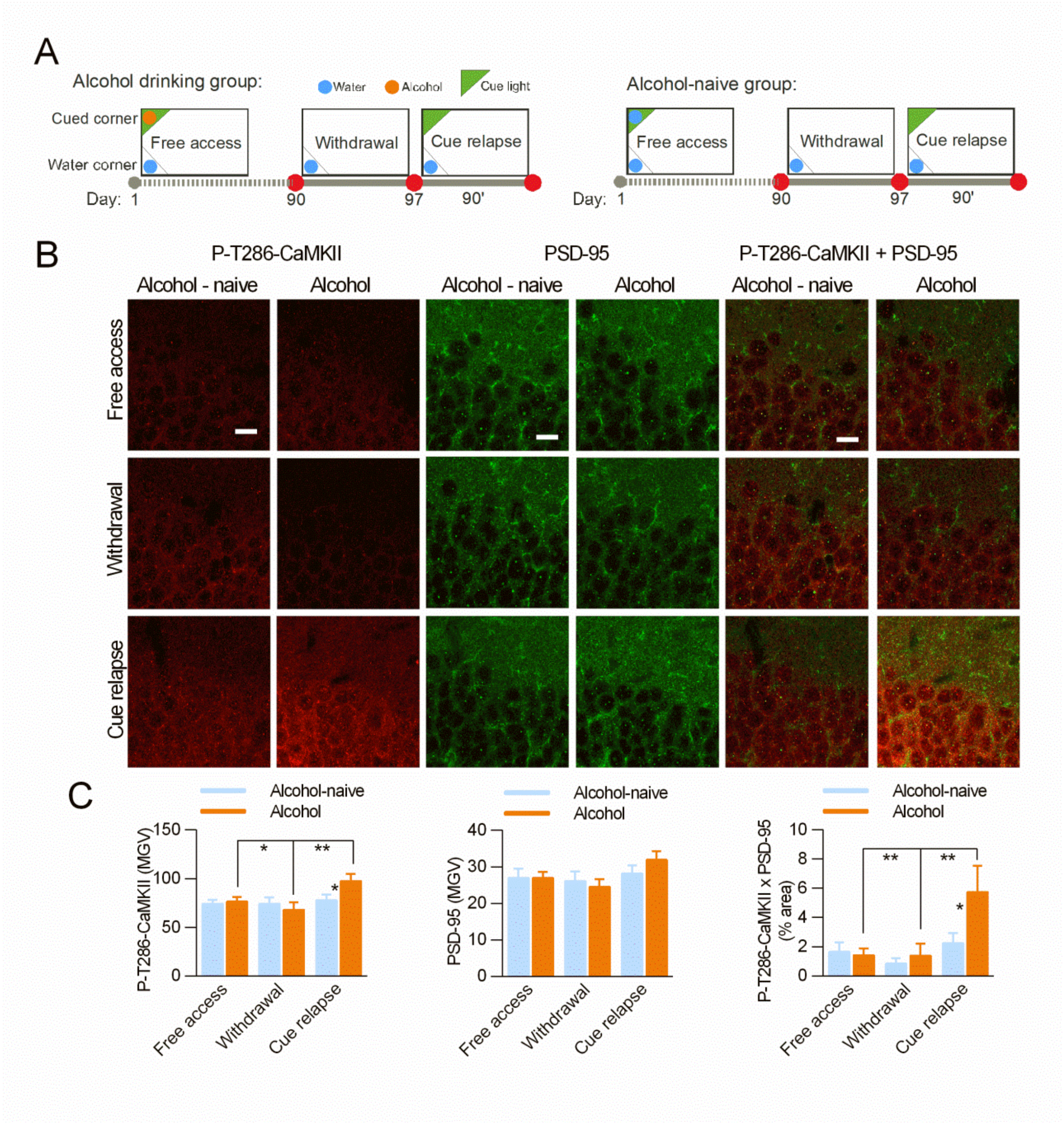

Suplementary Figure 2. Cue relapse incraeses the levels of P-T286-CaMKII in DG.

(A) Experimental timeline and IntelliCage setups for alcohol drinking and alcohol-naïve mice. Mice went through period of free access to alcohol (90 days), withdrawal (7 days) and cue relapse (90 min). Two groups of alcohol drinking (n=7) and naïve mice (n=7) were sacrificed after each stage of the training (black dots) (N=42). The brain section were prepared to perform immunostaining.

(B) Representative microphotographs of the upper blade (granular and molecular layer) of dorsal DG fluorescent immunostaining performed with specific antibodies recognizing autophosphorylated calcium and calmodulin-dependent kinase (P-T286-CaMKII) (used as a marker of ongoing synaptic plasticity (Giese et al. 1998; Shonesy et al. 2014; Coultrap et al. 2014) and synaptic marker, PSD95 (Broadhead et al. 2016). Scale bars, 100 μm.

(C) Summary of data showing that presentation of alcohol-, but not water-, associated cues increases the levels of P-T286-CaMKII (2-way ANOVA, training: $F(2, 33)=3.748$, $P=0.034$, alcohol: $F(1, 33)=1.008$, $P=0.322$), and colocalization of P-T286-CaMKII with PSD95 (training: $F(2, 32)=5.802$, $P=0.007$, alcohol: $F(1, 32)=2.996$, $P=0.093$), but has no effect on PSD95 expression (training: $F(2, 33)=2.141$, $P=0.133$; alcohol: $F(1, 33)=0.1427$, $P=0.708$) .*$p<0.05$, **$p<0.01$ by Tukey's multiple comparisons test

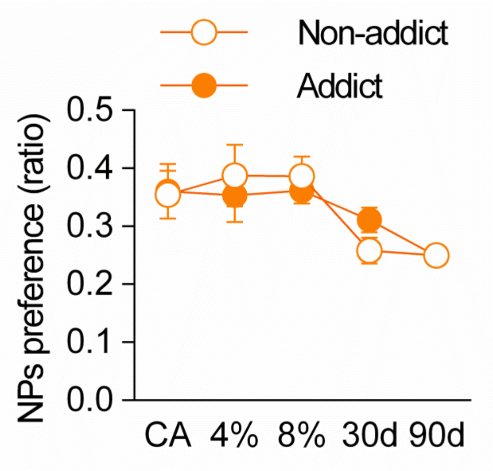

Supplementary Figure 3. Non-addict and addict mice do not differ in nose-poke preference for the reward corner. RM ANOVA, addiction index: F(1, 174)=0.0001, p=0.9900. CA, cage adaptation; 4% & 8%, alcohol initiation; 30d, the first 30 days of free access to alcohol; 90d, free access to alcohol, days 31-90.

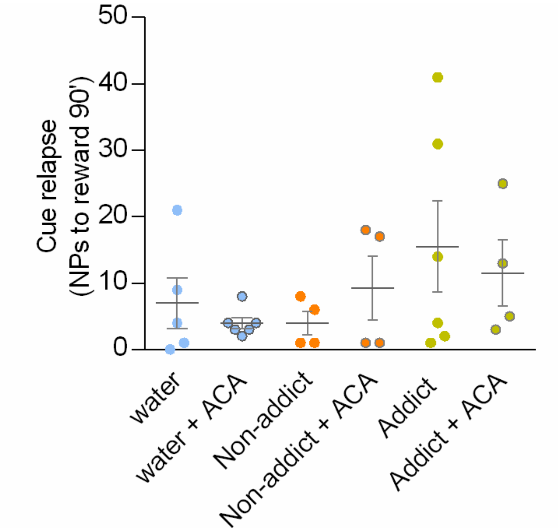

Supplementary Figure 4. Acamprosate does not affect alcohol seeking during initial 90 minutes of cue relapse. ACA, acamprosate. Kruskal-Wallis test, H=2.833, p=0.725.

Literature

Broadhead MJ, Horrocks MH, Zhu F, et al (2016) PSD95 nanoclusters are postsynaptic building blocks in hippocampus circuits. Sci Rep 6:24626 . doi: 10.1038/srep24626

Coultrap SJ, Freund RK, O'Leary H, et al (2014) Autonomous CaMKII mediates both LTP and LTD using a mechanism for differential substrate site selection. Cell Rep 6:431–7 . doi: 10.1016/j.celrep.2014.01.005

Giese KP, Fedorov NB, Filipkowski RK, Silva a J (1998) Autophosphorylation at Thr286 of the alpha calcium-calmodulin kinase II in LTP and learning. Science 279:870–873 . doi: 10.1126/science.279.5352.870

Shonesy BC, Jalan-Sakrikar N, Cavener VS, Colbran RJ (2014) CaMKII: A molecular substrate for synaptic plasticity and memory. Prog Mol Biol Transl Sci 122:61–87 . doi: 10.1016/B978-0-12-420170-5.00003-9
